# Elevated expression of LIF predicts a poor prognosis and promotes cell migration and invasion of clear cell renal cell carcinoma

**DOI:** 10.3389/fonc.2022.934128

**Published:** 2022-08-03

**Authors:** Wenting Zhong, Hongxia Liu, Feng Li, Youyu lin, Yan Ye, Luyun Xu, ShengZhao Li, Hui Chen, Chengcheng Li, Yuxuan Lin, Wei Zhuang, Yao Lin, Qingshui Wang

**Affiliations:** ^1^ Central Laboratory at the Second Affiliated Hospital of Fujian Traditional Chinese Medical University, Fujian-Macao Science and Technology Cooperation Base of Traditional Chinese Medicine-Oriented Chronic Disease Prevention and Treatment, Innovation and Transformation Center, Fujian University of Traditional Chinese Medicine, Fuzhou, China; ^2^ College of Life Sciences, Fujian Normal University, Fuzhou, China; ^3^ Department of Pathology, Fujian Provincial Hospital, Fuzhou, China; ^4^ Department of Urology, The Second Affiliated Hospital of Fujian Medical University, Quanzhou, China

**Keywords:** LIF, ccRCC, WGCNA, lasso, nomogram, invasion

## Abstract

**Background:**

Renal cell carcinoma (RCC) is the seventh most common cancer in humans, of which clear cell renal cell carcinoma (ccRCC) accounts for the majority. Recently, although there have been significant breakthroughs in the treatment of ccRCC, the prognosis of targeted therapy is still poor. Leukemia inhibitory factor (LIF) is a pleiotropic protein, which is overexpressed in many cancers and plays a carcinogenic role. In this study, we explored the expression and potential role of LIF in ccRCC.

**Methods:**

The expression levels and prognostic effects of the LIF gene in ccRCC were detected using TCGA, GEO, ICGC, and ArrayExpress databases. The function of LIF in ccRCC was investigated using a series of cell function approaches. LIF-related genes were identified by weighted gene correlation network analysis (WGCNA). GO and KEGG analyses were performed subsequently. Cox univariate and LASSO analyses were used to develop risk signatures based on LIF-related genes, and the prognostic model was validated in the ICGC and E-MTAB-1980 databases. Then, a nomogram model was constructed for survival prediction and validation of ccRCC patients. To further explore the drug sensitivity between LIF-related genes, we also conducted a drug sensitivity analysis based on the GDSC database.

**Results:**

The mRNA and protein expression levels of LIF were significantly increased in ccRCC patients. In addition, a high expression of LIF has a poor prognostic effect in ccRCC patients. LIF knockdown can inhibit the migration and invasion of ccRCC cells. By using WGCNA, 97 LIF-related genes in ccRCC were identified. Next, a prognostic risk prediction model including eight LIF-related genes (TOB2, MEPCE, LIF, RGS2, RND3, KLF6, RRP12, and SOCS3) was developed and validated. Survival analysis and ROC curve analysis indicated that the eight LIF-related-gene predictive model had good performance in evaluating patients’ prognosis in different subgroups of ccRCC.

**Conclusion:**

Our study revealed that LIF plays a carcinogenic role in ccRCC. In addition, we firstly integrated multiple LIF-related genes to set up a risk-predictive model. The model could accurately predict the prognosis of ccRCC, which offers clinical implications for risk stratification, drug screening, and therapeutic decision.

## Introduction

Renal cell carcinoma (RCC) is a malignant tumor that is derived from the lining of the proximal convoluted tubule (1). It accounts for approximately 90% of all renal malignancies, which has the highest mortality rate of the genitourinary system tumors (1). Based on genetic knowledge and histological findings, RCC is classified into five subtypes: common or conventional RCC (clear cell RCC); papillary RCC; chromophobe RCC; collecting duct carcinoma, with medullary carcinoma of the kidney; and unclassified RCC (2). In contrast to non-clear cell RCC (non-ccRCC), clear cell renal cell carcinoma (ccRCC) is the most common pathological subtype of renal cell cancer, which accounts for 75% of renal cell cancers (3). As ccRCC is not sensitive to chemotherapy and radiotherapy, surgical resection is the mainstay treatment at present. However, metastasis is a common event for ccRCC and 25% to 30% of patients have distant metastasis at the time of diagnosis (4). Several reports have demonstrated that the targeted therapies for ccRCC and non-ccRCC and their prognoses are quite different, and the prognosis of non-ccRCC is significantly better than that of ccRCC (5). Thus, further studies are required to clarify the underlying molecular mechanism of ccRCC progression and develop more efficient therapeutic targets for ccRCC.

Leukemia inhibitory factor (LIF) is a multifunctional cytokine belonging to the interleukin-6 superfamily, which was first reported in a study regarding M1 murine myeloid leukemic cells (6). LIF was initially defined by its ability to induce macrophage differentiation in M1 murine myeloid leukemic cells and inhibit their proliferation (7). Emerging evidence suggested that LIF plays an important and complex role in human cancers, although LIF has shown tumor-suppressive function in some types of cancers, including leukemia. LIF has been found to be overexpressed in more types of cancers in the past and has played a carcinogenic role. Currently, the detailed function of LIF in ccRCC has not been reported.

In the present study, we investigated the expression of the LIF gene and its potential role in renal cell carcinoma. In addition, a predictive model with prognostic significance based on LIF-related genes was constructed and validated. This study can lay a foundation for further research on the individualized treatment of ccRCC.

## Materials and methods

### Clinical data acquisition and extraction

The mRNA expression of LIF and clinical data for ccRCC patients were downloaded from The Cancer Genome Atlas (TCGA) database (https://www.cancer.gov/tcga). For TCGA dataset, RNA sequencing data (FPKM values) were normalized into log2 (FPKM + 1). The microarray datasets GSE15641, GSE46699, GSE53757, and GSE66272 were downloaded from the Gene Expression Omnibus (GEO) database (https://www.ncbi.nlm.nih.gov/geo/). The method for extracting microarray gene expression values is based on our previous research (8).

The gene expression data of ccRCC used for the validation cohort were obtained from the International Cancer Genome Consortium (ICGC) (https://icgc.org/) and ArrayExpress (https://www.ebi.ac.uk/arrayexpress). The accession number of ICGC is RECA-EU, including 91 who had follow-up information. The accession number of ArrayExpress is E-MTAB-1980, including 106 who had follow-up information.

### Patients with ccRCC recruitment

In the research, 30 ccRCC specimens from the patients of Fujian Provincial Hospital were selected. The study was performed with the approval of the Ethics Committee of Fujian Provincial Hospital and complied with the Helsinki Declaration. The written informed consent was obtained from all participating ccRCC patients.

### RT-PCR

TRIzol was performed to extract total RNA. Then, RNA was reverse transcribed with mRNA Reverse Transcription Kit (Takara, Japan). RT-PCR was performed using SYBR Green Kit (Vazyme, China). The primer sequences were shown as follows: LIF forward primer 5′-CTTGGCGGCAGGAGTTGT-3′, LIF reverse primer 5′-TTGTGACATGGGTGGCGTAT-3′; GAPDH forward primer 5′-GGAAGGACTCATGACCACAGTCC-3′; GAPDH reverse primer 5′-TCGCTGTTGAAGTCAGAGGAGACC-3′. GAPDH was used as the loading control. Gene expression levels were determined by the 2^-ΔΔCT^ method.

### Validation of protein expression of the LIF gene

The Human Protein Atlas (THPA) provides cell and tissue distribution information for 26,000 human proteins. It uses specific antibodies to identify protein expression in tumor tissues and normal tissues. In the research, we explored the protein expression of the LIF gene in ccRCC tissues and normal tissues.

### Weighted gene correlation network analysis

Weighted gene correlation network analysis (WGCNA) is a common algorithm used to build gene co-expression networks (9). The WGCNA R package was employed to execute WGCNA analysis. A power of β = 6 and a scale-free R (2) = 0.87 were selected as soft-threshold parameters to ensure a signed scale-free co-expression gene network. A cluster dendrogram was created based on the topological overlap matrix with a minimum cluster size of 20.

### Gene ontology and kyoto encyclopedia of genes and genomes pathway enrichment analyses

Gene Ontology (GO) analysis and Kyoto Encyclopedia of Genes and Genomes (KEGG) pathway enrichment were calculated by functional enrichment tool DAVID. DAVID bioinformatics resources provide an integrated biological database and a repository of analytic tools for systematic exploration of the biological meaning of gene set DAVID. The default parameters in the tool were used, and enriched pathways were ranked according to their enrichment scores. A *p*-value of <0.05 was identified as enriched functions.

### LASSO analysis

The Least Absolute Shrinkage and Selection Operator (LASSO) was used to construct an LIF-related-gene risk-predictive model with the help of “survival” and “glmnet” packages in R software. LASSO is a common method used in high-dimensional data regression, which can select prognosis-related gene pairs of ccRCC by shrinking regression coefficients. The optimal penalty weight of the Lasso–Cox model was found in a grid search manner in a 10-fold cross-validation process. Then, the coefficients of most gene pairs were reduced to zero, and a small number of gene pairs with non-zero coefficients were closely correlated with the prognosis of ccRCC.

### Cell lines, cell culture, and transfection

The ccRCC cell lines 786-O and ACHN cells were obtained from the American Type Culture Collection (ATCC, Manassas, VA, USA). 786-O cells and ACHN cells were respectively cultured in PRMI 1640 (Gibco by Life Technologies, Grand Island, NY, USA) and DMEM (Gibco by Life Technologies, Grand Island, NY, USA) containing 10% fetal bovine serum (FBS, BI, Kibbutz Beit Haemek, Israel) at 37°C in a humidified incubator with 5% CO_2_. The sequences of shRNA1 and shRNA2 targeting LIF were respectively cloned into pLVX vectors. The following shRNA sequences were used: LIF shRNA-1, 5′-GGGTAAGGATGTCTTCCAGAA-3′; LIF shRNA-2, 5′-GGAAGTATAAGCAGATCATCG-3′. The PEI transfection system (Invitrogen) was used for transfection according to the manufacturer’s guidelines.

### Cell counting kit-8 assay

Cell proliferation was detected by Cell Counting Kit-8 (CCK-8) assay. 786-O cells and ACHN cells were prepared into cell suspension with a density of 1 × 10^4^ cells/ml, respectively. Cell suspension (0.2 ml) was added to four 96-well plates (2 × 10^3^ cells/well) and cultured in a 5% CO_2_ incubator at 37°C. The culture was repeated for 24, 48, and 72 h. Ten microliters of CCK-8 solution was added in 1 h before measuring the absorbance. After incubation, the absorbance was measured at 450 nm with a microplate reader.

### Scratch assay

The transfected 786-O cells and ACHN cells were inoculated on six-well plates (1 × 10^6^ cells/well), respectively. The cells were serum-starved for this assay to avoid the effects of cell viability. When cell convergence reached ~90%, a scratch was made in the monolayer with the tip of a 10-µl pipette perpendicular to the bottom of the hole, which was then washed with PBS twice to remove unattached cells. Images were taken at 0 and 24 h, of which 0 h was recorded as the starting point. The cells were photographed for evaluation of wound closure under an inverted microscope. ImageJ software was used to process the image and calculate the migration area. The following formula was used: Change rate of scratch area (%) = (0 h scratch area-24 h scratch area)/0 h scratch area.

### Transwell assay

Cell migration assay was performed using an 8-μm cell culture insert (Falcon). Cell invasion was examined using polycarbonate membrane Transwell inserts (Costar; Corning Inc.). After 48 h of transfection, cells were added to the upper chamber (2 × 10^4^ cells/well) with 200 µl serum-free medium. The upper chamber was incubated for 24 h in a 24-well plate chamber with 200 µl complete medium containing 10% FBS. After the cells at the top of the upper cavity were wiped with a cotton swab, the transplanted cells at the bottom of the cavity were stained in 0.1% crystal violet solution at room temperature for 10 min. The migrated and invading cells were photographed under an inverted microscope and counted in four random fields.

### Single-cell analysis

CancerSEA depicts single-cell functional status maps that contain 14 functional states obtained from 41,900 individual cells (http://biocc.hrbmu.edu.cn/CancerSEA/) (10). In the study, CancerSEA was used to evaluate the potential roles of LIF genes in ccRCC.

### DepMap

The Cancer Dependency Map (https://depmap.org/portal/) developed CERES to estimate gene-dependency levels on the survival of cells (11, 12). Dependency scores for the LIF gene in ccRCC cells were calculated using CERES.

### Drug sensitivity evaluation

GSCALite is a website used for drug sensitivity analysis (http://bioinfo.life.hust.edu.cn/web/GSCALite/). In the study, we used the GSCALite database to evaluate the drug sensitivity of LIF-related genes to identify potential molecular compounds for targeted therapy.

### Statistical analysis

The statistical analysis was evaluated by t-test in this study. Paired samples used the paired t-test, and unpaired samples used the unpaired t-test. Correlations between LIF expression and clinicopathological characteristics were performed by the chi-squared test. The Kaplan–Meier method was used for overall survival (OS), and the log-rank test was used for comparison of survival curves. Cox regression analysis was performed for univariate and multivariate survival analyses. All *p* values smaller than 0.05 were considered to be significantly different from each control.

## Results

### High expression of LIF was correlated with poor prognosis of ccRCC

**Figure 1:**
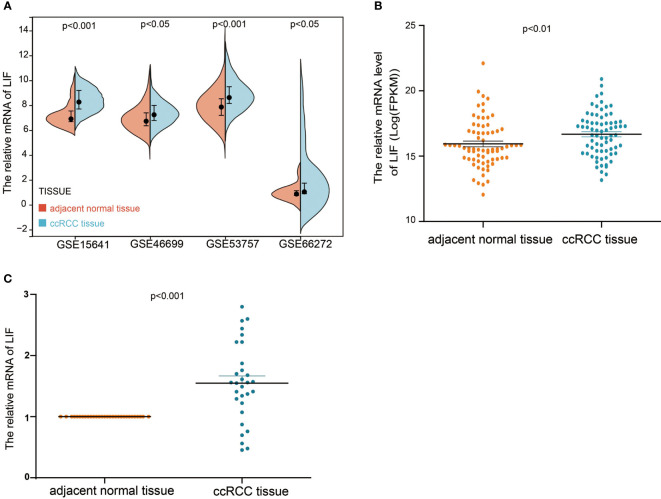
The mRNA expression of LIF in ccRCC. **(A)** The mRNA expression of LIF for normal tissues and ccRCC tissues in GSE15641, GSE46699, GSE5375, and GSE6627. **(B)** The mRNA expression of LIF for ccRCC patients in TCGA database. **(C)** The mRNA expression of LIF for ccRCC patients in 30 ccRCC patients.

In order to evaluate the mRNA expression of LIF mRNA in ccRCC, paired renal carcinoma and paracancer tissues obtained from TCGA and GEO databases, including GSE15641, GSE46699, GSE53757, and GSE66272, were selected for analysis. GSE15641 consisted of 23 pairs of adjacent normal kidney tissue and ccRCC tissue. GSE46699 consisted of 63 pairs of adjacent normal kidney tissue and ccRCC tissue. GSE53757 consisted of 72 pairs of adjacent normal kidney tissue and ccRCC tissue. GSE66272 consisted of 27 pairs of adjacent normal kidney tissue and ccRCC tissue. As shown in **Figures 1A,B**, mRNA levels in ccRCC tissues were significantly upregulated compared with normal kidney tissues (GSE15641, *p* < 0.001; GSE46699, *p* < 0.05; GSE53757, *p* < 0.001; GSE66272, *p* < 0.05; TCGA, *p* < 0.001). In TCGA database, 263 and 264 patients were in LIF low and LIF high expression groups, respectively. The results showed that the LIF high expression group had remarkably more patients with stage T III and IV (*p* = 0.001) and stage IIIandIV (*p* = 0.046) than the LIF low expression group (**Table 1**). Meanwhile, we measured LIF mRNA levels in 30 clinical ccRCC samples using quantitative RT-PCR. The results showed that the expressions of LIF in ccRCC tissues were significantly higher than those in adjacent tissues (*p* < 0.001; **Figure 1C**).

**Table 1:** Characteristics of ccRCC patients and their LIF expression level.

Characteristics	Low level of LIF (N = 263)	High level of LIF (N = 264)	Total (N = 527)	*p* value
**Age**				0.76
>60	130 (24.67%)	135 (25.62%)	265 (50.28%)	
≤60	133 (25.24%)	129 (24.48%)	262 (49.72%)	
**Gender**				0.45
Male	166 (31.50%)	176 (33.40%)	342 (64.90%)	
Female	97 (18.41%)	88 (16.70%)	185 (35.10%)	
**Pathologic M**				0.06
M1and4	45 (8.57%)	63 (12.00%)	108 (20.57%)	
M0	218 (41.52%)	199 (37.90%)	417 (79.43%)	
**Pathologic N**				0.24
N1and4	138 (26.19%)	153 (29.03%)	291 (55.22%)	
N0	125 (23.72%)	111 (21.06%)	236 (44.78%)	
**Pathologic T**				0.03
T1and2	81 (15.37%)	107 (20.30%)	188 (35.67%)	
T3and4	182 (34.54%)	157 (29.79%)	339 (64.33%)	
**Stage**				0.03
Stage I and II	88 (16.79%)	115 (21.95%)	203 (38.74%)	
Stage III and IV	172 (32.82%)	149 (28.44%)	321 (61.26%)	

**Figure 2:**
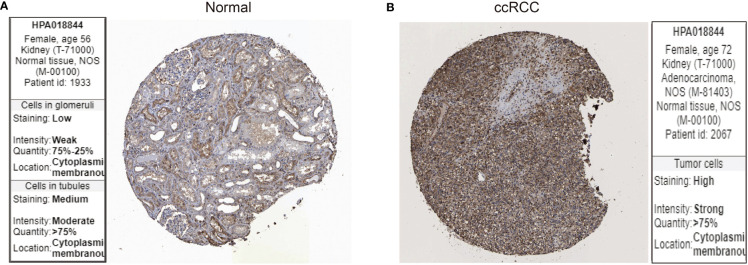
The protein expression of LIF in ccRCC. The LIF protein expression in normal tissues **(A)** and ccRCC tissues **(B)** was analyzed through the Human Protein Atlas database.

Next, we examined the protein expression for LIF using immunohistochemistry images from the Human Protein Atlas database. In specimens from normal human kidney tissue, immunohistochemical examination revealed that LIF protein was weakly expressed in the glomeruli and moderately in the tubules (**Figure 2A**), while LIF protein was strongly expressed in ccRCC tissues (**Figure 2B**). Collectively, these results implicated that the mRNA and protein expression levels of LIF in ccRCC tissues were higher than those in normal tissues.

**Figure 3:**
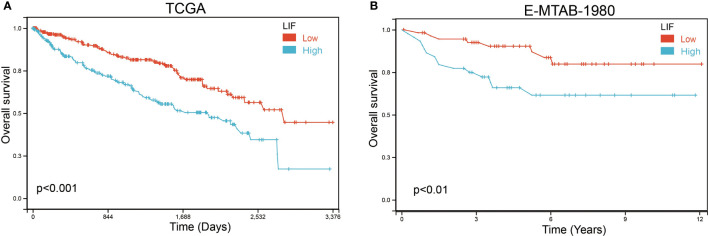
The OS of LIF for ccRCC patients. Overall analysis for the prognostic value of LIF expression for OS in ccRCC patients by Kaplan–Meier analysis based on TCGA **(A)** and E-MTAB-1980 **(B)**. The Kaplan–Meier method was used to draw survival curves.

Based on Kaplan–Meier survival analysis, we evaluated the overall survival (OS) of ccRCC patients to explore the clinical significance of LIF. The results demonstrated that a high LIF expression correlated with poor prognosis in ccRCC patients based on TCGA and E-MTAB-1980 databases (**Figure 3**).

Univariate and multivariate analyses were performed to determine the predictors for OS. In univariate analysis, high expression levels of LIF, stage IIIandIV, stage T3and4, stage M1and4, stage N0, and age >60 were revealed to be associated with a poor OS rate of patients with ccRCC. Furthermore, to evaluate the independent impact of the LIF expression level on OS, a multivariate Cox’s regression model was performed. The results demonstrated that a high LIF expression level was a poor independent prognostic factor for OS in patients with ccRCC. In addition, stage M1and4, stage III and IV, and age >60 revealed independent prognostic value in the multivariate analysis (**Table 2**).

**Table 2:** Univariate and multivariate Cox proportional hazard model for OS in ccRCC patients based on TCGA database.

Variables	Univariate		Multivariate	
	HR (95% CI)	*p*	HR (95% CI)	*p*
**Age**				
≤60	Reference	<0.001	Reference	<0.01
>60	1.75 (1.27-2.44)		1.56 (1.12-2.17)	
**Gender**				
Male	Reference	>0.05		
Female	0.95 (0.68-1.32)			
**Pathologic M**				
M0	Reference	<0.001	Reference	<0.001
M1and4	4.07 (2.94-5.62)		2.21 (1.50-3.25)	
**Pathologic N**				
N0	Reference	<0.05	Reference	>0.05
N1and4	0.81 (0.59-1.11)		0.77 (0.56-1.07)	
**Pathologic T**				
T1and2	Reference	<0.001	Reference	>0.05
T3and4	3.62 (2.62-5.00)		1.01 (0.52-1.96)	
**Stage**				
Stage I and II	Reference	<0.001	Reference	<0.01
Stage III and IV	4.33 (3.08-6.08)		2.95 (1.40-6.23)	
**LIF expression**				
Low	Reference		Reference	
High	2.06 (1.50-2.84)	<0.001	1.86 (1.34-2.58)	<0.01

### Knockdown of LIF suppressed migration and invasion of ccRCC cells

**Figure 4:**
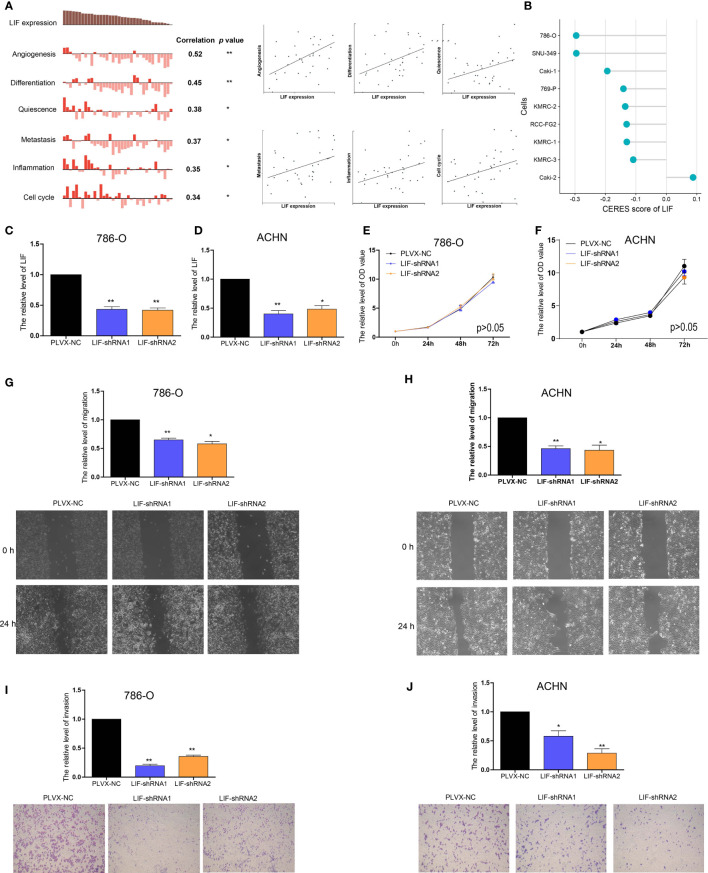
The role of LIF in ccRCC cells **(A)** Data from CancerSEA demonstrated that LIF mRNA expression was positively correlated with angiogenesis, differentiation, quiescence, metastasis, inflammation, and cell cycle. **(B)** Gene effect scores of LIF in ccRCC cells from RNAi and CRISPR/Cas9 screens. **(C, D)** The LIF expression changes was confirmed by real-time PCR in the 786-O **(C)** and ACHN **(D)** cells after transfecting LIF-shRNAs. **(E, F)** The proliferation ability of 786-O **(E)** and ACHN **(F)** cells were measured after transfecting LIF-shRNAs. (GandH) The migration ability of 786-O **(G)** and ACHN **(H)** cells was measured after transfecting LIF-shRNAs. (IandJ) The invasion ability of 786-O **(I)** and ACHN **(J)** cells was measured after transfecting LIF-shRNAs. *p < 0.05; **p < 0.01.

In order to further investigate the functions of LIF in ccRCC, CancerSEA was used to determine whether LIF was related to the carcinogenic process in ccRCC, and it was found that the functional phenotype of LIF was positively related to angiogenesis, differentiation, quiescence, metastasis, inflammation, and cell cycle (**Figure 4A**). DepMap is a website used to identify genes critical for the survival and proliferation of tumor cells. A negative score for CERES indicates that the knockout gene inhibits tumor cell survival and proliferation, while a positive score indicates that the knockout gene promotes survival and proliferation. A CERES score <-1 was defined as an essential gene for tumor cell survival. From the DepMap website, we obtained the CERES scores of nine ccRCC cell lines. The CERES scores of LIF in nine ccRCC cell lines ranged between -0.296 and 0.1, and the mean CERES score was -0.149 (**Figure 4B**). These results indicated that LIF might influence ccRCC cell metastasis but has no effect on proliferation.

Using RT-PCR, we found that the expression of LIF mRNA was significantly higher in 786-O and ACHN ccRCC cell lines than in the HEK-293 normal renal cell line (**Supplementary Figure 1**). Then, 786-O cells and ACHN cells were transfected with LIF-shRNA1 and LIF-shRNA2 to knock down LIF expression (**Figures 4C, D**). Compared with the corresponding negative control, CCK8 assay indicated that the effect of LIF knockout on the proliferation of 786-O cells and ACHN cells was not significant (**Figures 4E, F**). Cell migration assay showed that LIF knockdown significantly arrested the migration of 786-O cells and ACHN cells (**Figure 4G, H**). Additionally, the Transwell assay indicated that LIF knockdown inhibited the invasion abilities of 786-O cells and ACHN cells (**Figures 4I, J**). In summary, LIF promotes the migration and invasion abilities of ccRCC cells.

### Identification of key modules and co-expression-related genes of LIF

**Figure 5:**
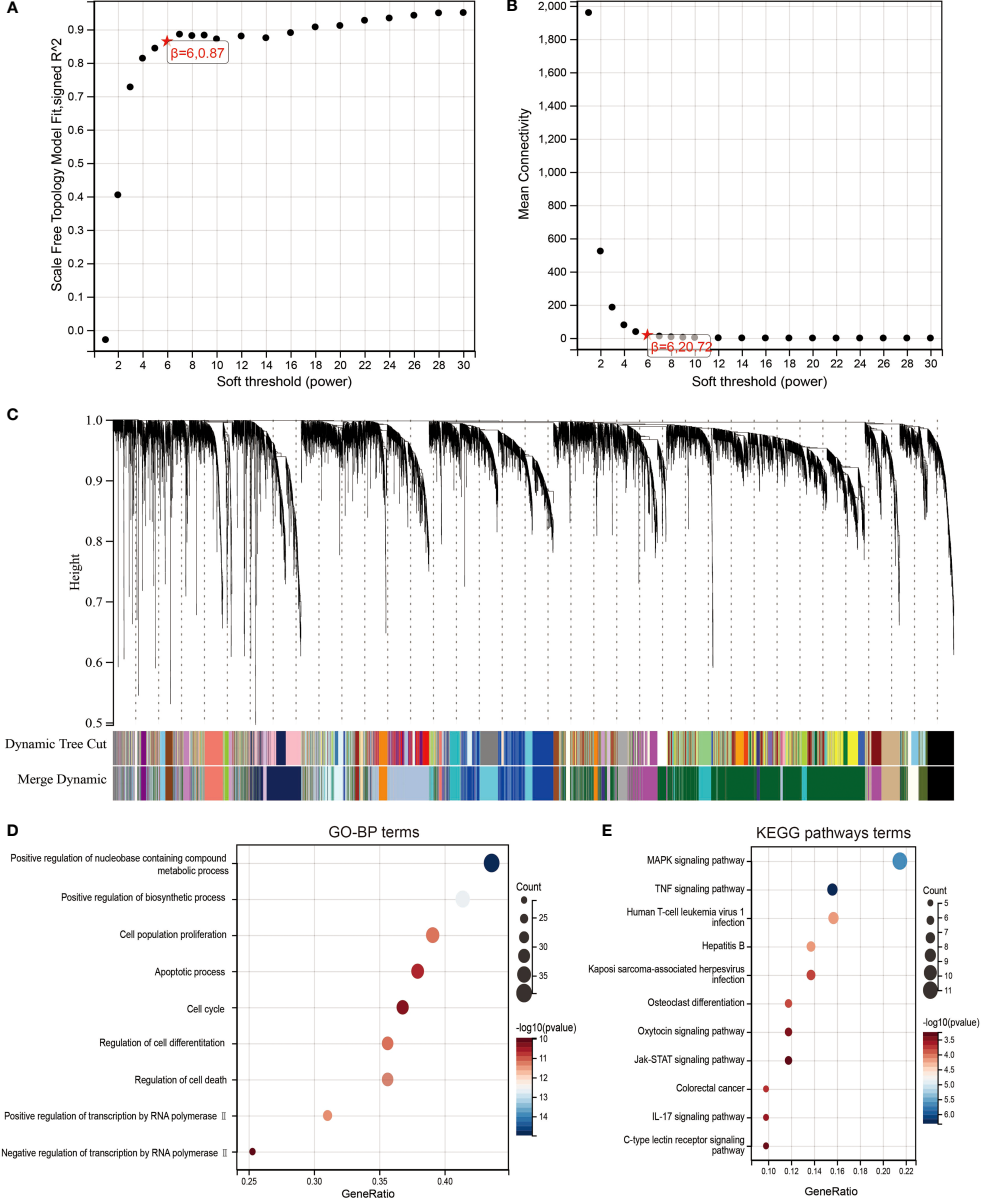
Identification of co-expression module genes associated with LIF using the WGCNA. **(A)** Relationship between scale-free topology model fit and soft thresholds (powers). **(B)** Relationship between the mean connectivity and various soft thresholds. **(C)** Dendrogram of modules identified by WGCNA. (DandE) GO-BP **(D)** and KEGG pathway **(E)** network for the target genes in green model.

To identify highly associated genes with LIF in ccRCC patients, we constructed a gene co-expression network using the “WGCNA” R package. Database from TCGA was used to build WGCNA. We calculated the network topology for soft-thresholding powers from 1 to 30 to choose the best threshold. A power of β = 6 and minimum module size = 20 were set as per the standard scale-free networks (**Figures 5A, B**). Dynamic Tree Cut represents the original module, while Merged Dynamic means the final module. Dynamic Tree Cut yielded a total of 38 modules with different colors identified. Among them, the violet module is the gene co-expressed with LIF, which contains 97 genes (**Figure 5C**).

To clearly determine biological processes and cellular pathways dependent on the Violet module, we used the DAVID functional classification tool to analyze Gene Ontology (GO) and KEGG pathways (**Figures 5D,E**). The terms of biological processes (BP) were positive regulation of nucleobase, containing compound metabolic process, positive regulation of biosynthetic process, cell population proliferation, apoptotic process, cell cycle, regulation of cell differentiation, regulation of cell death, positive regulation of transcription by RNA polymerase II, and negative regulation of transcription by RNA polymerase II. The terms of KEGG pathway terms were MAPK signaling pathway, TNF signaling pathway, human T-cell leukemia virus 1 infection, hepatitis B, Kaposi sarcoma-associated herpesvirus infection, osteoclast differentiation, oxytocin signaling pathway, Jak-STAT signaling pathway, colorectal cancer, IL-17 signaling pathway, and C-type lectin receptor signaling pathway.

### Prognosis model of LIF-related-gene construction and validation

**Figure 6:**
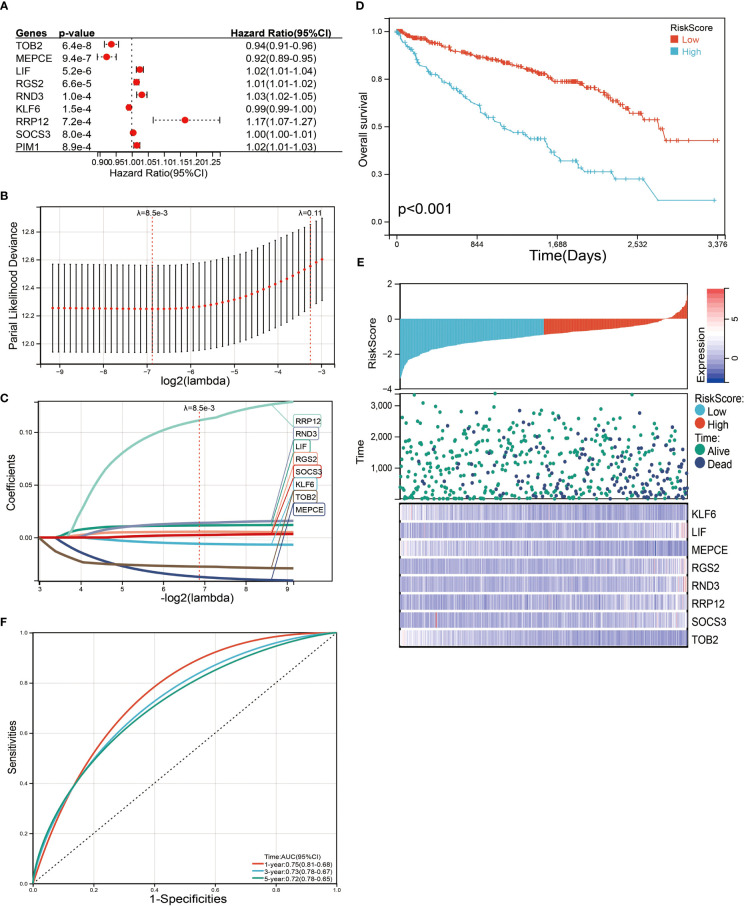
Identification and screening of the prognosis-related genes. **(A)** Univariate Cox regression analysis was used to assess the genes that related to prognosis. **(B)** Partial likelihood deviance of OS for the LASSO coefficient profiles. **(C)** LASSO coefficient profiles of the TOB2, MEPCE, LIF, RGS2, RND3, KLF6, RRP12, and SOCS3 expression for OS. **(D)** Kaplan–Meier curves to compare overall survival of low-risk and high-risk groups. **(E)** The distribution of risk score, survival status, and mRNA expression levels of ccRCC patients in TCGA database. **(F)** ROC curves compare the prognostic accuracy of the classifier in ccRCC patients using AUCs at 1, 3, and 5 years to assess prognostic accuracy.

**Figure 7:**
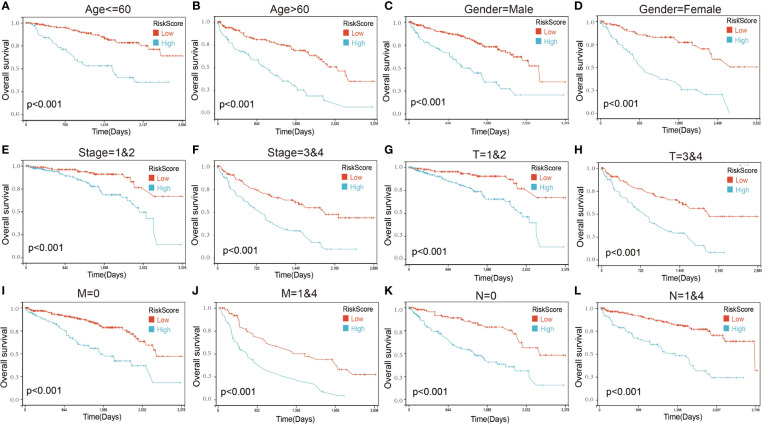
Kaplan–Maier survival curves of overall survival of ccRCC patients according to risk score model in different subgroups. **(A, B)** Prognosis analysis of the ccRCC patients with age ≤60 **(A)** and age >60 **(B)** subgroup. **(C, D)** Prognosis analysis of the ccRCC patients with gender = male **(C)** and gender = female **(D)** subgroup. **(E, F)** Prognosis analysis of the ccRCC patients with stage = 1and2 **(E)** and stage = 3and4 **(F)** subgroup. **(G, H)** Prognosis analysis of the ccRCC patients with T = 1and2 **(G)** and T = 3and4 **(H)** subgroup. **(I, J)** Prognosis analysis of the ccRCC patients with M = 0 **(I)** and M = 1and4 **(J)** subgroup. **(K, L)** Prognosis analysis of the ccRCC patients with N = 0 **(K)** and N = 1and4 **(L)** subgroup. The Kaplan–Meier method was used to draw survival curves.

We next performed a univariate Cox survival analysis on these 97 LIF-related genes. Furthermore, the results indicated that nine LIF-related genes were associated with the prognosis of ccRCC patients. The high levels of six LIF-related genes (LIF, RGS2, RND3, RRP12, SOCS3, and PIM3) were significantly correlated with shorter OS for ccRCC patients, whereas the remaining three LIF-related genes (TOB2, MEPCE, and KLF6) were significantly associated with longer OS for ccRCC patients (**Figure 6A**). The results of the expression analysis showed that the expressions of KLF6, RND3, and SOCS3 in ccRCC tissues were significantly higher compared with those in adjacent normal tissues, whereas the expressions of RGS2 and TOB2 were significantly reduced in ccRCC tissues. However, the expressions of PIM1 and MEPCE were not significantly different between ccRCC tissues and adjacent normal tissues (**Supplementary Figure 2**). It has already been demonstrated that multiple genes can better predict patient prognosis. Thus, we ran the LASSO-Cox regression model and calculated the regression coefficient based on the aforementioned nine LIF-related genes. Cross-validation was applied to overcome the overfitting effect, and the optimal λ value of 0.0085 was selected (**Figure 6B**). An ensemble of eight genes remained with their individual LASSO coefficients, and the distribution of LASSO coefficients of the gene signature is shown in **Figure 6C**. Risk score = (0.1112 * RRP12) + (0.0138 * RND3) + (0.0114 * LIF) + (0.0054 * RGS2) + (0.0023 * SOCS3) + (-0.0059 * KLF6) + (-0.0276 * TOB2) + (-0.0374 * MEPCE). Each ccRCC patient was divided into high-risk and low-risk groups according to the risk score. The Kaplan–Meier curve analysis result showed that the high-risk group correlated with the poor prognosis of ccRCC patients (**Figure 6**). The distribution of risk score, survival time, and the LIF-related gene level of ccRCC patients in TCGA database are shown in **Figure 6E**. The ROC analysis demonstrated that the LIF-related risk score had a powerful ability to predict OS in ccRCC patients (1 year (AUC = 0.75), 3 years (AUC = 0.72), 5 years (AUC = 0.72); (**Figure 6**). To further assess the robustness of the risk score model, we stratified the ccRCC population based on age, gender, stage, and TMN. After stratification of age ≤60 (**Figure 7A**), age >60 (**Figure 7B**), gender = male (**Figure 7C**), gender = female (**Figure 7D**), stage = 1and2 (**Figure 7E**), stage = 3and4 (**Figure 7F**), stage T = 1and2 (**Figure 7G**), stage T = 3and4 (**Figure 7H**), stage M = 0 (**Figure 7I**), stage M = 1and4 (**Figure 7J**), stage N = 0 (**Figure 7K**), and stage N = 1and4 (**Figure 7L**), respectively, the risk score based on the eight-mRNA signature was an independent prognostic indicator, and patients with high risk scores had a poorer prognosis. These results further confirmed the relatively good stratification ability of the prognostic model.

**Figure 8:**
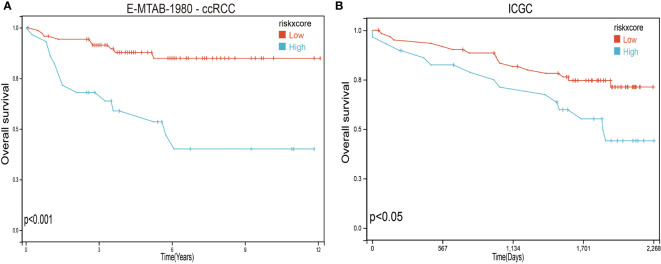
Validation of the prognosis risk model The prognosis risk model was validated using E-MTAB-1980-ccRCC **(A)** and ICGC **(B)** databases.

To further verify the validity and stability of the prognostic model, we respectively downloaded 101 and 91 samples with complete clinical information from the E-MTAB-1980-ccRCC database and the ICGC database. Each patient was brought into the previous prognostic model to calculate the risk score. Patients were divided into high-risk and low-risk groups. Kaplan–Meier curve analysis showed that ccRCC patients with low-risk scores had a better OS than those in the high-risk-score group (**Figure 8**), indicating good accuracy.

### Construction of a clinical prognostic prediction model

**Figure 9:**
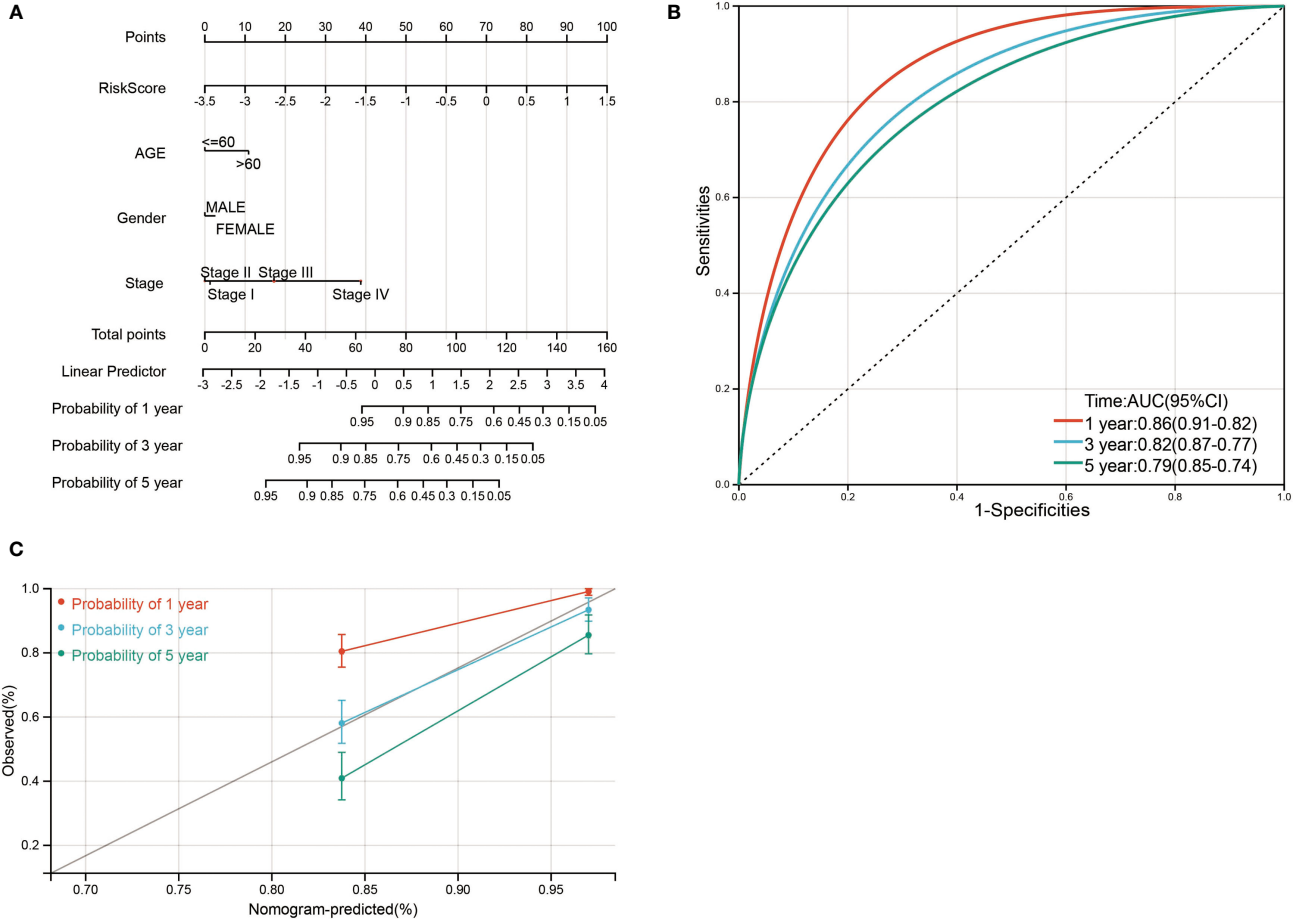
Nomogram risk prediction and validation of ccRCC patients based on risk score. **(A)** Nomogram for predicting 1-, 3-, and 5-year events that combine clinical data with age, gender, stage, and risk score. The line segment corresponding to each variable is marked with a scale, which represents the value range of the variable, and the length of the line segment reflects the contribution of the factor to the outcome event. The point in the figure represents the individual score corresponding to each variable under different values, and the total score of the corresponding individual scores after all variables was taken. **(B)** ROC curve was used to verify the diagnosis with AUC at 1, 3, and 5 years. **(C)** The validation plots for predicting overall survival.

Finally, a nomogram was constructed by incorporating four prognostic indicators from the database, including age, sex, stage, and risk score, into the final model. In the nomogram, the probability of survival at 1, 3, and 5 years in this particular population was as shown in **Figure 9A**. The ROC curve was used to verify the diagnostic effect, and AUC was found to be greater than 0.7 regardless if it is 1 year (AUC, 0.86; 95% CI, 0.91–0.82), 3 years (AUC, 0.82; 95% CI, 0.87–0.77), and 5 years (AUC, 0.79; 95% CI, 0.85–0.74) (**Figure 9B**), suggesting that this nomogram was reliable and robust. Calibration plots showed excellent calibration of the nomogram (c-index 0.79) (**Figure 9C**). We hold the opinion that the nomogram may have good accuracy for survival prediction in ccRCC patients.

### The relationship of LIF-related genes and drug sensitivity

**Figure 10:**
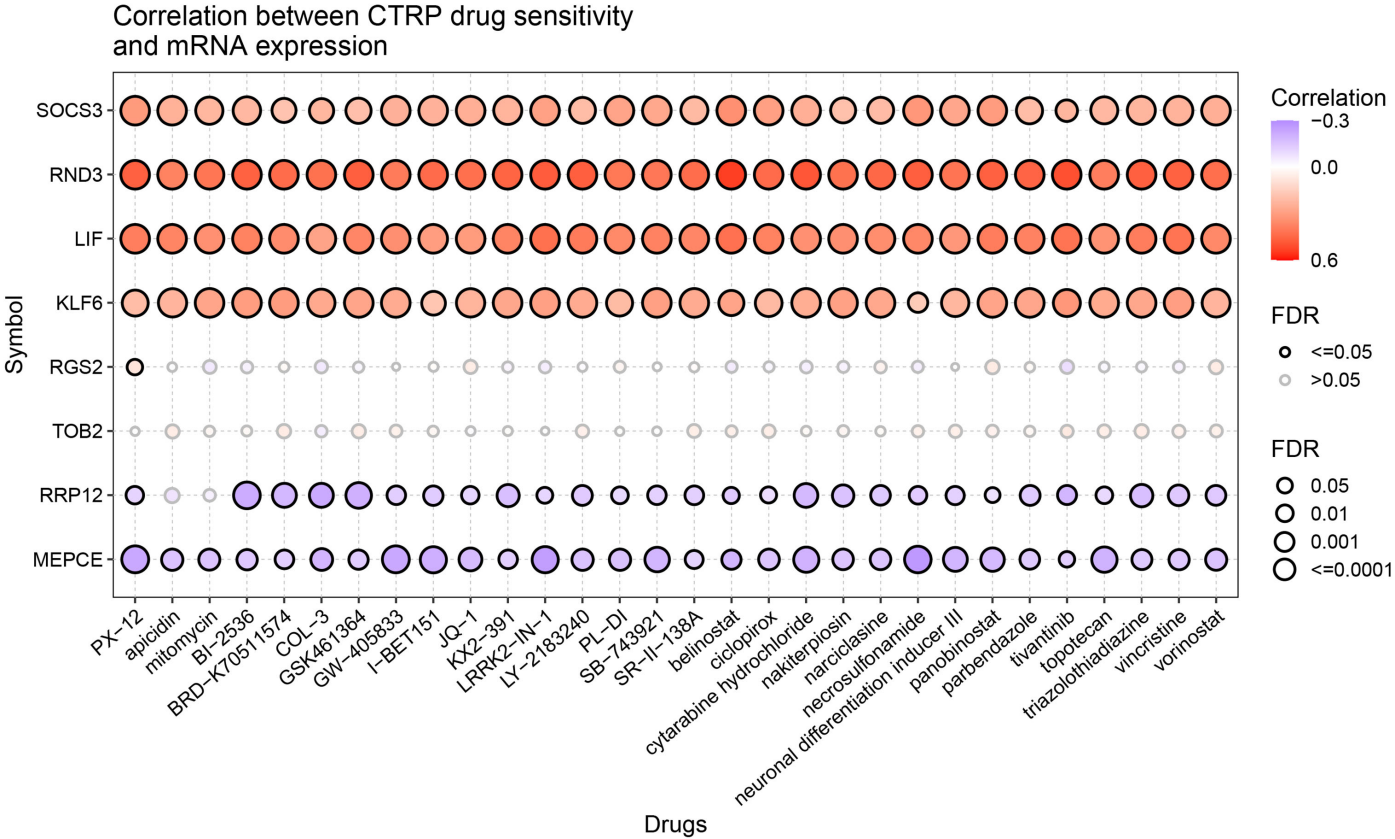
Drug sensitivity of the LIF-related genes from CTRP. The correlations between the LIF-related gene expression and drugs. The positive Spearman correlation coefficients indicate that high gene expression is resistant to the drug, and vice versa.

The relationship between LIF-related genes and drug sensitivity was explored based on the data from the Cancer Therapeutics Response Portal (CTRP) database by using the GSCA website. High LIF, SOCS3, RND3, and KLF6 expressions were associated with higher drug resistance to PX-12, apicidin, mitomycin, BI-2536, and vorinostat, etc. (**Figure 10**).

## Discussion

LIF is a multifunctional cytokine that affects cell growth by inhibiting differentiation (13, 14). LIF is involved in a number of key processes in cancer growth and progression, including immune tolerance (15), chemotherapy (16) and radiation (17) resistance, maintenance of cancer stem cell-like phenotypes, and EMT (18). Targeting LIF has been actively investigated as a novel strategy for cancer therapy (19). Many studies have proved that LIF plays an important role in breast cancer (20), pancreatic cancer (21), gastric cancer (22), and ovarian cancer (23). For instance, LIF promotes the proliferation, invasion, and metastasis of breast cancer. This promotion occurs through the activation of AKT, which activates the downstream mTOR signaling pathway (24). At the same time, LIF was reversed to promote tumor formation and metastasis (25). However, the role of LIF in ccRCC has not been studied.

In this study, we observed the mRNA and protein of LIF to be highly expressed in ccRCC, and a high expression of LIF was associated with poor prognosis in ccRCC patients. Functional experiments revealed that LIF knockdown did not affect ccRCC cell growth, and suppressed migration and invasion of ccRCC cells was observed. It indicates that the malignant potential of LIF in ccRCC is reflected in promoting tumor migration.

Next, we analyzed the genes highly associated with LIF in ccRCC patients by WGCNA and found that these 97 LIF-related genes were enriched in MAPK signaling pathway, JAK/STAT signaling pathway, and so on. Recent studies have shown that LIF can selectively activate a variety of signaling pathways, including JAK/STAT (26), PI3K/Akt (27), MAPK (28), and mTOR (24), depending on cell type and tissue-specific modalities. The JAK/STAT pathway was originally defined as a signal transduction pathway downstream of the cytokine receptor, which is involved in many important biological processes such as cell proliferation, differentiation, apoptosis, and immune regulation (29). The JAK family consists of four members: JAK1, JAK2, JAK3, and TYK2 (30). Experiments using overexpressed components initially showed that LIF receptors bind to at least three members of the JAK family (JAK1, JAK2, and TYK2). Activation kinetics of JAK1 was found to be significantly faster after LIF exposure compared to JAK2 and TYK2, again suggesting that it was the kinase initially targeted by LIF (31). Activation of JAK1 catalyzed phosphorylation of tyrosine residues on the receptor, and these phosphorylated tyrosine sites and surrounding amino acid sequences formed docking sites to which STAT proteins containing the SH2 domain were recruited. STAT3 was considered to be the most important signal sensor after LIF stimulation and mediates most cellular effects (32). STAT3 docks to phosphorylated tyrosines in both the gp130 and LIFRβ chains of the LIF receptor at YxxM motifs (33). JAK1 catalyzes phosphorylation of STAT3 protein bound to the receptor, and the activated STAT protein enters the nucleus in the form of dimer to bind to target genes and regulate gene transcription.

The MAPK signaling pathway is a basic pathway in mammalian cells, which is closely related to physiological activities such as cell proliferation, differentiation, apoptosis, and angiogenesis. Studies have shown that an abnormal activation of certain proteins in the MAPK pathway is an important cause of many cancers. MAPK is an evolutionarily conserved group of silk/threonine protein kinases that, similar to STAT activation, are activated by activated LIF receptors (34). Two chains of the LIF receptor, GP130 and LIFR, both contain phosphorylation sites that recruit SHP2 (35). Activated SHP2 induces Ras/Raf signaling pathways, which in turn activate MAPK and eventually transcriptional activators such as ELK to transmit signals from the cell membrane to the nucleus (36). Even if the MAPK signaling is induced by LIF, it obeys STAT3 and PI3K signaling in this system (28).

Using Cox univariate analysis and Lasso regression, we constructed a prognostic risk model including eight LIF-related genes (RRP12, RND3, LIF, RGS2, SOCS3, KLF6 TOB2, and MEPCE). To assess the reliability of the risk prognostic model, we conducted external validation and subgroup analysis. The AUC values of the ROC curves of the 1-, 3-, and 5-year survival of the model were all greater than 0.7, which indicated that the signature composed of eight LIF-related genes had good performance in predicting the prognosis of ccRCC.

To date, only SOCS3 has been reported to be regulated by LIF. SOCS3 is an inducible negative feedback inhibitor of cytokine signaling and widely reported to be regulated by LIF.

RRP12 is an RNA-binding protein mainly involved in the extranuclear transport of the 40S and 60S subunit precursors of ribosomes. RRP12 regulates yeast cell cycle and DNA damage response (37). In osteosarcoma cells, RRP12 can enhance cell resistance to chemotherapy drugs, and p53 expression was significantly upregulated after interfering RRP12 expression (38). RRP12 knockout can inhibit the proliferation, invasion, and metastasis of HCC (39) and GC cells.

RND3/RhoE is an atypical member of the Rho-Gtpase family (40) and is involved in functions normally regulated by rho-GTPase as well as many basic cellular processes. The regulation of Rnd3 varies in different cancers. RND3 is under-expressed in gastric cancer (41), hepatocellular carcinoma (42), and prostate cancer (43), while it is overexpressed in pancreatic cancer (44) and non-small cell lung cancer (45).

There have been many reports on the regulation of RGS2 in tumors, and the abnormal expression of RGS2 can be seen in a variety of tumors, such as colon cancer (46), ovarian cancer (47), and prostate cancer (48). RGS2 inhibits the growth of melanoma cells by inhibiting MAPK and AKT. RGS2 inhibits melanoma cell growth by inhibiting MAPK and AKT, but this effect depends on the genetic structure of the overall cell (49).

KLF6, a gene that encodes a zinc finger DNA-binding transcription factor, is one of the strongest superenhancers in ccRCC cells (50). In addition, KLF6 has both growth-suppressive and supportive functions in different cancers. For instance, overexpression of KLF6-SV1 in prostate cancer cell lines leads to increased proliferation (51).

The human Tob proteins (Tob1 and Tob2) are encoded by paralogous genes belonging to the mammalian BTG/Tob family of anti-proliferative factors that regulate cell growth in a variety of cell types (52, 53). In human primary HCCs, inhibition of Tob2 reproduces mir-362-3p overexpression, thereby increasing cell proliferation and anchorage-independent soft agar growth (54). In adipose tissue, Tob2 negatively regulates adipogenesis by inhibiting PPARγ2 expression (55).

7SK RNP was associated with high elongation of RNA polymerase II. The basic structure of 7SK RNP includes LARP7, MEPCE, and 7SK RNA. 7SK RNA has a special 5-terminal phosphate monomethyl cap catalyzed by a phosphomethylation capping enzyme (MePCE) (56), which is also a stationary component of 7SK RNP. In addition, MEPCE is also involved in miRNA targeting and regulation. MEPCE, for example, has been identified as the targeting and negative regulation of Mir-338, which is associated with the migration and invasion of HCC cells (57).

Additionally, we performed a drug sensitivity analysis on LIF-related genes and found that LIF, RND3, SOCS3, and KLF6 have closely related sensitivity to numerous drugs. These findings provide a new reference for drug treatment of ccRCC.

However, our study had limits that should be acknowledged. We conducted and validated the LIF-related prognostic risk mode by utilizing general databases, and the outcomes require in-depth confirmation by prospective research. In future work, studies to clarify the specific mechanisms of LIF in ccRCC are warranted.

## Conclusion

In summary, high expression of LIF is an important factor for poor prognosis of ccRCC patients. Inhibition of LIF can suppress the migration of ccRCC cells. The risk score model including LIF-related genes can be used to predict the prognosis of ccRCC patients, leading to improved monitoring of the present patient population.

## Data availability statement

The original contributions presented in the study are included in the article/**Supplementary Material**. Further inquiries can be directed to the corresponding author/s.

## Ethics statement

This study was approved by the Research Ethics Committee of Fujian Provincial Hospital and complied with the Helsinki Declaration. Written consent was obtained from all study participants.

## Author contributions

WTZ and QW contributed to the conception and design. HL, FL, YoL, YY, LX, SL, HC, CL, and YuL contributed to the development of methodology. WZ, YaL, and QW contributed to the writing, review, and/or revision of the manuscript. All authors contributed to the article and approved the submitted version.

## Funding

This research was funded by the National Natural Science Foundation of China (82003095), Natural Science Foundation of Fujian Province (2022J01173, 2022J01273, 2022J011015, and 2020J01311402), and Young and Middle-Aged Talent Training Project in Fujian Provincial Health System (2020GGA008).

## Conflict of interest

The authors declare that the research was conducted in the absence of any commercial or financial relationships that could be construed as a potential conflict of interest.

## Publisher’s note

All claims expressed in this article are solely those of the authors and do not necessarily represent those of their affiliated organizations, or those of the publisher, the editors and the reviewers. Any product that may be evaluated in this article, or claim that may be made by its manufacturer, is not guaranteed or endorsed by the publisher.

## Supplementary material

Supplementary Figure 1 The expression of LIF in HEK-293, 786-O and ACHN cell.

Supplementary Figure 2 The heat map analysis of LIF related genes based on TCGA database.
