# Supplementary figures and images for: Elevated expression of LIF predicts a poor prognosis and promotes cell migration and invasion of clear cell renal cell carcinoma
Source: Front Oncol. 2022 Aug 3;12:934128. doi: 10.3389/fonc.2022.934128 (PMC9382297; doi:10.3389/fonc.2022.934128)

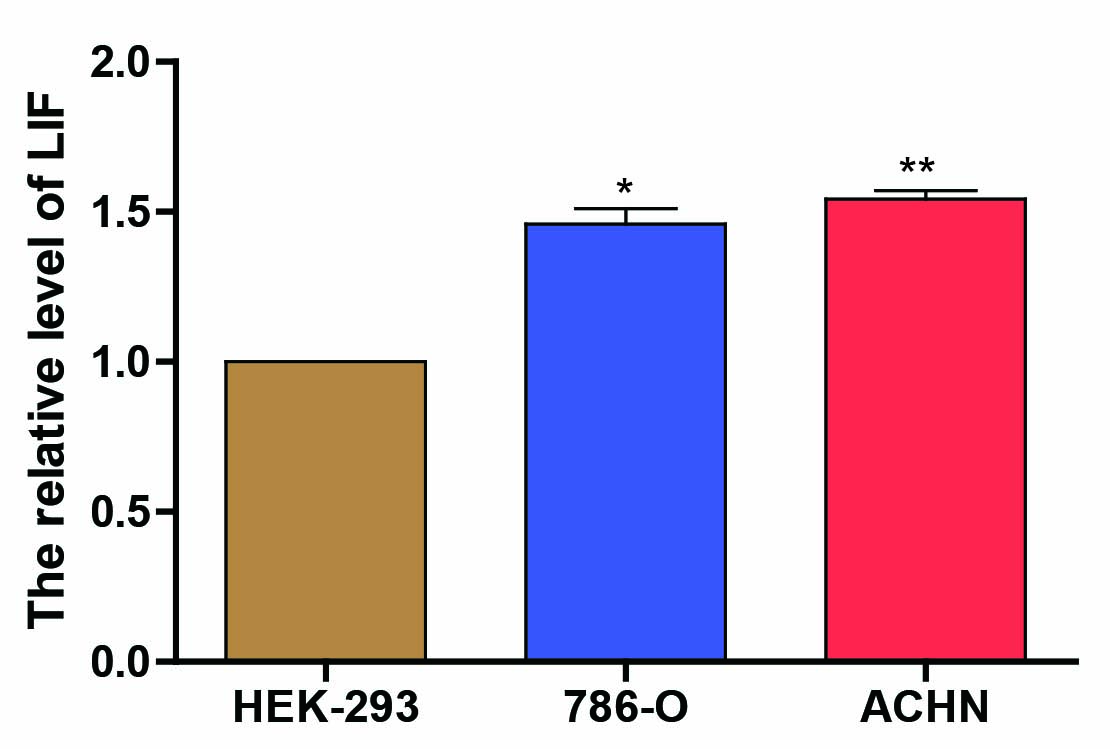

Supplement: Supplementary Figure 1 — The expression of LIF in HEK-293, 786-O and ACHN cell. [file Image_1.jpeg]

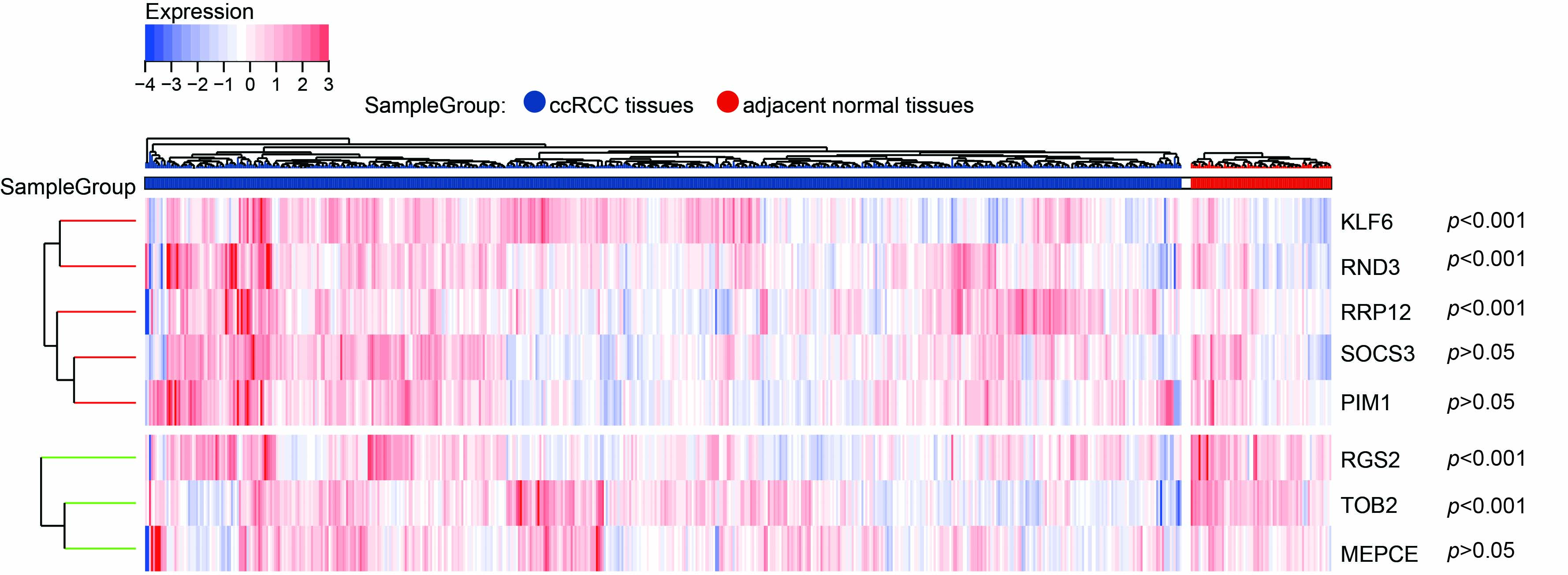

Supplement: Supplementary Figure 2 — The heat map analysis of LIF related genes based on TCGA database. [file Image_2.jpeg]
